# Modulating Vimentin: A Systems-Level Therapeutic Strategy for Sepsis and Complex Diseases

**DOI:** 10.3390/life16030457

**Published:** 2026-03-11

**Authors:** Ruihuan Chen, Jianping Wu, Daniel Jafari, Annica K. B. Gad

**Affiliations:** 1 Aluda Pharmaceuticals, Inc., Union City, CA 94587, USA; 2 Laboratory Animal Center, School of Medicine & Holistic Integrative Medicine, Nanjing University of Chinese Medicine, Nanjing 210029, China; wujianping@njucm.edu.cn; 3 Northwell, New Hyde Park, NY 11040, USA; djafari@northwell.edu; 4 Department of Oncology-Pathology, Karolinska Institutet, 171 64 Solna, Sweden; annica.gad.2@ki.se; 5 School of Science and Technology, Örebro University, 701 82 Örebro, Sweden

**Keywords:** sepsis, vimentin, systems biology, complex system, host response dysregulation, network coordination, therapeutic strategy, disease-modifying therapy

## Abstract

Sepsis remains a leading global health challenge, characterized by high mortality and a persistent lack of disease-modifying therapies. Despite decades of investment, therapeutic progress has been constrained by reductionist strategies that target isolated pathogenic components. This perspective argues that these failures reflect a fundamental mischaracterization of sepsis—not as a disorder of discrete pathways, but as the collapse of complex biological systems in which normally coordinated processes become desynchronized. We identify the intermediate filament protein vimentin as a determinant of system fate governing the transition from adaptive host defense to pathological breakdown. Acting as a context-dependent network integrator and signal amplifier, vimentin coordinates antagonistic cellular programs by integrating biochemical and biophysical cues across immune, vascular, and metabolic systems. Under physiological stress, this coordination enables the orderly activation and resolution of inflammatory and suppressive responses required for pathogen control and restoration of homeostasis. In sepsis, persistent or excessive insults drive vimentin-mediated overactivation, uncoupling these programs and propagating systems-level instability that culminates in organ dysfunction. By integrating mechanistic, preclinical, and emerging clinical evidence, this perspective proposes vimentin modulation as a clinically translatable, systems-oriented strategy aimed at realigning host response networks to address the dynamic, opposing pathologies of sepsis that have eluded current therapies.

## 1. Introduction

Sepsis continues to pose a major challenge to global health systems, affecting approximately 49 million individuals and claiming 11 million lives annually [1]. In the United States alone, sepsis accounts for at least 350,000 adult deaths each year [2,3], exceeding the combined mortality of the six deadliest cancer types [4]. Despite this urgency, no sepsis-specific therapy beyond standard care has reduced mortality in more than three decades [5].

This persistent failure underscores the complex nature of sepsis as a catastrophic desynchronization of interdependent biological systems. Characterized by the concurrent escalation of opposing processes, including hyperinflammation and immunosuppression, hyper- and hypocoagulability, and catabolism alongside anabolism [6,7,8,9,10], sepsis produces chaotic clinical trajectories that defy reductionist assessment [11,12,13]. Effective treatment therefore requires mechanisms capable of resolving this complexity through the dynamic and simultaneous modulation of divergent pathogenic pathways.

Vimentin, one of the most evolutionarily conserved intermediate filament proteins in vertebrates [14], has emerged as the ideal candidate for this systemic role. Far more than a mere structural scaffold, vimentin functions as a central network hub and mechano-transducer, integrating diverse signals that sustain health or drive disease [15,16,17]. Its role is intrinsically bidirectional and spans the primary domains of sepsis pathology: it modulates immune stimulatory and suppressive cell activities [18,19], regulates endothelial integrity and leakage [20,21], and orchestrates coagulation [22,23], mitochondrial dynamics [24,25], and metabolism [26,27]. When these tightly regulated mechanisms become imbalanced or uncoupled, as occurs in sepsis, the system shifts into a state of chaotic, self-amplifying destruction. Vimentin’s broad regulatory reach across these divergent pathways therefore positions it as a unique systems-level therapeutic target.

Just as the complexity of sepsis has historically been difficult to unravel, the versatile, conflicting functions of vimentin have been equally challenging to reconcile. In this perspective, we adopt a systems biology framework to characterize sepsis as a collapsing complex system, arguing that drug development must transcend reductionism in favor of systems-oriented strategies. By delineating the central systems-level role of vimentin, we present conceptual models defining the evolutionary logic of vimentin and revealing how its adaptive mechanisms shift host defense from protective resilience to systemic failure. Further, we synthesize mechanistic, preclinical and emerging clinical evidence to establish vimentin as a biologically robust and clinically actionable therapeutic target for the dynamic, opposing pathologies of sepsis.

## 2. Sepsis as a Catastrophic Failure of Complex Systems

Complexity theory offers a powerful framework for decoding the chaotic nature of sepsis, reframing the disorder not as a linear sequence of events but as a system-wide collapse [11,28].

### 2.1. The Features of Complex Systems

Biological organisms, along with their constituent parts, such as cells, tissues and organs, function as complex systems composed of numerous interconnected and interdependent components. Their collective interactions generate *emergent behaviors* that cannot be inferred from individual parts alone, reflecting the system’s inherent *adaptivity* and *nonlinearity,* in which minor perturbations can yield disproportionately large effects, while major inputs may yield minimal impact, rendering outcomes *unpredictable*. These systems *self-organize* without centralized control, relying on amplifying and dampening *feedback loops* to maintain *resilience*: the ability to preserve integrity while adapting to changing conditions. This dynamic interplay sustains the organism in a zone between rigid order and uncontrolled disorder, i.e., the *‘edge of chaos’*, which defines the critical thresholds beyond which the system drives toward either **Rigid Failure** or **Chaotic Collapse** [29].

### 2.2. The Mechanics of Collapse: From Disequilibrium to Uncoupling

In dynamic environments, resilience requires the rapid neutralization of threats through a temporary, controlled departure from equilibrium. Upon detecting an insult, the system initiates a stimulatory response to neutralize the threat, momentarily destabilizing internal homeostasis. To prevent runaway activation, intrinsic inhibitory mechanisms are subsequently triggered to counterbalance this surge. The coordinated engagement of these opposing forces, stimulation and suppression, defines the system’s capacity and resilience to withstand stress.

Under physiological conditions, successful threat neutralization allows these counteracting forces to dampen one another, guiding the system back to baseline. However, if the external threat persists, stimulatory mechanisms become self-reinforcing, overwhelming inhibitory control. This drives the system into a state of sustained, high-tension disequilibrium. As the magnitude of these opposing forces escalates, stability erodes. Once a critical threshold is breached, the regulatory coordination fractures. This catastrophic uncoupling marks the transition from an adaptive stress response to systemic failure, characterized by cascading breakdowns and emergent maladaptive behaviors [30].

### 2.3. The Sepsis Paradigm: Uncoupling of Antagonistic Forces

Sepsis represents the clinical manifestation of this systemic collapse. Here, delayed pathogen clearance instigates a self-reinforcing immunostimulatory response, driving profound hyperinflammation. In a desperate bid to restore homeostasis, the system engages compensatory immunoinhibitory mechanisms. However, under persistent infection, these antagonistic forces are relentlessly amplified until regulatory synchronization is lost [31].

**Figure 1:**
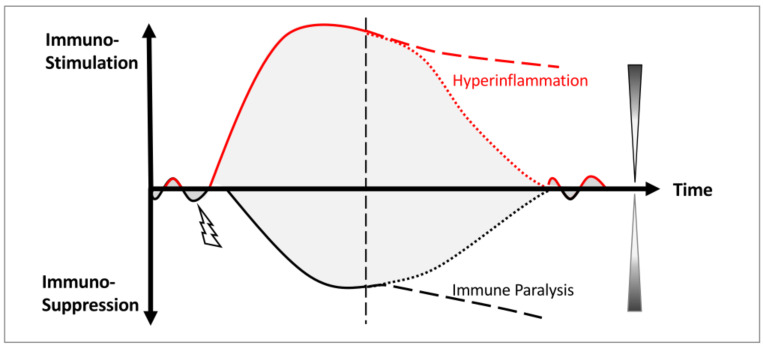
Summative model of sepsis progression: from excessive functional amplification to the pathological uncoupling of immune stimulation and compensatory immune suppression. Immune stimulation (top left axis) and immune suppression (bottom left axis), and infection (lightning symbol). Gradient triangles (right) indicate the relative contribution of immune stimulation (top) and suppression (bottom) to the system disequilibrium. Immunostimulatory (redlines), compensatory immunosuppressive response (black lines), hyperinflammation (dashed red line), immune paralysis (dashed black line), the tipping point of the system (vertical dashed line), and the recovery (dotted lines). Grey shaded regions denote the magnitude of coordinated immune response over time.

Consequently, rather than restoring homeostasis, these intensified responses become uncoupled (Figure 1). Hyperinflammation and immunosuppression escalate independently and concurrently, driving divergent, opposing pathologies [7,13,32]. This profound immune dysregulation propagates across organ systems, culminating in the chaotic, intractable clinical trajectories that define the progression of sepsis.

## 3. Vimentin as the On-Demand Amplifier of Host Defense

### 3.1. The Evolutionary Paradox: Non-Essential in Health, Critical in Crisis and Detrimental in Disease

#### 3.1.1. Conserved, Abundant and Widely Expressed

Vimentin is exceptionally conserved in vertebrates and abundantly expressed across diverse cell types. Sequence alignment reveals more than 97% amino acid identity among mammalian species from rodents to primates and humans, with nearly 100% conservation of the structural core (rod domain) that mediates filament assembly across species. Proteomic analyses estimate that vimentin can reach over 20 million copies per cell, ranking among the most abundantly expressed proteins [33]. Vimentin is the first type III intermediate filament to be expressed during development. It is highly expressed in immune cells and cells of mesenchymal origin and is strongly upregulated in epithelial cells undergoing epithelial-to-mesenchymal transition (EMT) [15,16,34].

#### 3.1.2. The Conundrum

The evolutionary conservation, high abundance and wide distribution of vimentin imply its functional importance. However, vimentin is surprisingly not essential for murine embryonic development; vimentin-deficient animals develop and reproduce without an overt phenotype [35]. This paradox, termed “the conundrum of the intermediate filaments” [36], has puzzled researchers for decades. Although vimentin appears largely dispensable in animals under well controlled environments, this dispensability vanishes under stress. When challenged by trauma, infection, or mechanical strain, vimentin-deficient systems exhibit significant functional deficits [15,34]. This dichotomy suggests that vimentin’s primary function is not to maintain basal homeostasis, but to reinforce systemic resilience during adversity.

While vimentin provides the resilience necessary to sustain health in a complex, dynamic environment, it paradoxically drives diverse pathologies in disease states. Consequently, vimentin depletion confers broad protective effects in animals. Vimentin knockout (KO) animals exhibit resistance to bacterial and viral infections [37,38], reduced renal and pulmonary fibrosis [39,40,41], and diminished glial scarring following spinal cord injury [42]. Furthermore, vimentin deficiency limits cancer metastasis [43], attenuates the severity of DSS-induced colitis [44], mitigates graft-versus-host disease (GVHD) after allogeneic transplantation [18], and improves survival in LPS-induced sepsis model [19]. To understand the complex biology of vimentin, and to establish the rationale for therapeutic intervention, it is essential to unravel the mechanisms that govern the functional versatility and the transition from a guardian of resilience to a driver of pathology.

### 3.2. The Structural Logic: A Dynamic Hub for Signal Integration

#### 3.2.1. Structural Plasticity and Functional Diversity

Vimentin’s functional versatility stems from its unique combination of biochemical and biophysical properties. Vimentin is extensively modified post-translationally, most notably by phosphorylation, oxidation, citrullination, O-GlcNAc glycosylation, acetylation, and proteolytic cleavage, which collectively regulate its assembly dynamics, enhance its conformational flexibility, and expand its structural and functional diversity [45,46,47].

Vimentin filaments themselves are structurally flexible, constantly assembling and disassembling to form context-dependent scaffolds [48,49,50]. This intrinsic structural plasticity enables adoption of diverse conformations, supporting interactions with a broad spectrum of binding partners and allowing vimentin to function as a central network hub that sequesters or presents signaling molecules in response to environmental cues [16,51,52]. Although vimentin lacks intrinsic enzymatic activity, its large and highly adaptable interactome, which shifts substantially across cell types, activation states, and environmental conditions, allows it to modulate diverse signaling pathways among different systems in health and in disease [15,45].

#### 3.2.2. Exceptional Binding Capacity

Various proteins have been identified to bind or interact with vimentin, including the following:

Bacterial components for facilitating infectivity, such as InlF (*Listeria monocytogenes*), IbeA (*Escherichia coli* K1), BspC (*Streptococcus agalactiae*/Group B Streptococcus), and IpaC (*Shigella flexneri*) [37];

Viral factors for supporting viral entry and replication, such as Spike (S) Protein (*SARS-CoV* and *SARS-CoV-2*), NS4B (*Dengue Virus*), Envelope (E) Protein (*Dengue Virus* & *Japanese Encephalitis Virus*), VP1 (*Enterovirus 71*), Vif (*HIV-1*), Nucleocapsid (N) Protein (*PRRSV*), and Capsid Proteins (*Cowpea Mosaic Virus*) [38,53];

Immune and inflammatory regulators, such as NLRP3 (Inflammasome) [19], p47phox (NADPH Oxidase) [44], Beclin-1 (Autophagy) [54], and HSP90 (Heat Shock Protein) [55];

Cytoskeletal and adhesion integrators, such as Fimbrin/Filamin A (Actin Crosslinkers) [56], Kinesin/Dynein (Microtubule Motors) [57], Plectin 1b (A cytolinker) [58], Scribble (A Polarity Protein) [59] and Integrins (adhesion molecules) [60];

Organelle tethers, such as mitochondria (directly or indirectly via Plectin) [24,61], FTCD/GORAB (Golgi Complex) [62,63], and AP-3 Complex (Lysosomes) [64];

Signaling molecules and transducers, such as 14-3-3 Proteins (signaling adaptors) [52], p47phox (regulatory protein for NADPH oxidase) [44], ERK (MAPK pathway) [65], GEF-H1 (RhoA activator) [66], and AKT1 (protein kinase of Rho family) [67].

Through these diverse interactions, vimentin serves as a signaling platform or scaffold that facilitates or restrains many biological processes based on the dominant input of the system. While this allows for rapid adaptation, it also creates a critical vulnerability: if upstream or initiating signals are hijacked by pathogens or dysregulated by persistent stress, vimentin faithfully amplifies these errors, transforming a defensive mechanism into a driver of disease.

### 3.3. Evidence of Bidirectional Amplification: Across Different System Levels—From Cell to Tissue

Vimentin’s regulation of cell contractility and tissue repair exemplifies its opposing roles in fine-tuning host responses to the environment at the cellular and tissue levels, respectively.

#### 3.3.1. Cellular Mechanics: An Adaptive Rheostat

Cellular traction forces are fundamental to the migration, adhesion, and mechano-sensing of cells. Cellular contractility has been suggested to be governed by a tensegrity-based force balance: actomyosin networks generate inward tension, while microtubules act as rigid compression struts to resist this force [68]. Vimentin interacts directly and indirectly with both actomyosin and microtubule networks to reinforce their functions. In cells on soft substrates, the actomyosin forms a weak network, generating low traction forces that exert minimal compressive load on microtubules. Under these conditions, vimentin’s role in reinforcing these weak actomyosin structures becomes dominant, effectively increasing traction forces to enhance cellular contractility; consequently, vimentin depletion on compliant matrices markedly reduces force generation, leaving cells with suboptimal contractility [69]. In contrast, on stiff matrices, actomyosin forms a robust network, generating high tensile forces that actively compress microtubules. Here, vimentin’s contribution to further actomyosin reinforcement becomes negligible; instead, its role in providing external structural support to microtubules under substantial compressive stress becomes paramount, thereby preventing excessive cellular contractility in stiff environments. Vimentin depletion in this context removes this essential bracing, rendering microtubules vulnerable to buckling. Without this mechanical buffer to resist and absorb tension, the system becomes less capable of restraining contractile forces, precipitating an unnecessary increase in cell contractility [69].

Therefore, at the cellular level, vimentin does not merely “increase” or “decrease” contractility in a linear fashion; instead, it acts as a dynamic rheostat that tunes cellular forces to meet environmental demands by selectively reinforcing antagonistic cytoskeletal elements. At the molecular level, this selectivity is dictated by the context-dependent mechanical states of actomyosin and microtubules across varying environments, rather than a shift in vimentin’s fundamental function, which is to consistently reinforce and amplify both counteracting mechanisms. By providing this universal reinforcement, vimentin exerts its most profound impact on the specific structural component currently most vulnerable to external stress, thereby ensuring the cell can achieve an adaptive cellular contractility.

#### 3.3.2. Tissue Repair Dynamics: An Accelerator and a Braker

During tissue repair, vimentin supports critical processes including cell migration, cytoskeletal remodeling, and fibroblast-mediated contraction [70]. However, its role appears regulatory rather than foundational. In excisional wound model, vimentin knockout (KO) mice exhibit a distinct biphasic healing trajectory: while early-phase healing is indistinguishable from wild-type (WT) controls, KO mice show delayed progression in the mid-phase followed by accelerated recovery in the late phase, ultimately achieving comparable closure times [71]. This pattern is mirrored in *in vitro* scrape-wound assays, where wound closure in KO condition displays initial parity with WT, followed by significant slowing during the progression phase and marked acceleration during resolution [71]. The delayed mid-phase suggests a failure in repair amplification, while the accelerated late-phase implies a loss of regulatory ‘braking’ signals.

Collectively, these findings indicate that while vimentin is not required for the initiation or completion of tissue repair, it is critical for calibrating the magnitude and temporal dynamics of the response. Vimentin appears to reinforce both positive and negative feedback mechanisms at distinct stages of tissue repair process, thereby accelerating repair progression while promoting controlled and coordinated resolution.

#### 3.3.3. Systemic Gain Control: Vimentin as an On-Demand Universal Amplifier

Viewed through a systems biology lens, vimentin has evolved not as a dedicated driver of any single pathway but as an *on-demand universal signal amplifier*. It increases the gain of whatever biological programs are active, stimulatory or inhibitory, thereby reinforcing what the system interprets as most in need.

Under homeostatic conditions, vimentin remains largely quiescent, an idle yet ready platform poised to preserve system integrity in responding to external perturbations. When a threat is detected, vimentin is rapidly mobilized to potentiate the relevant defensive response. However, this same amplifying architecture carries inherent risk: if the system misjudges the threat, vimentin intensifies wrong signals and propagates pathology; and when a threat cannot be promptly resolved, sustained vimentin-driven amplification can push cellular and systemic networks beyond their tolerance thresholds, ultimately precipitating collapse.

### 3.4. Conceptual Model: From Physiological Facilitator to Pathological Driver

**Figure 2:**
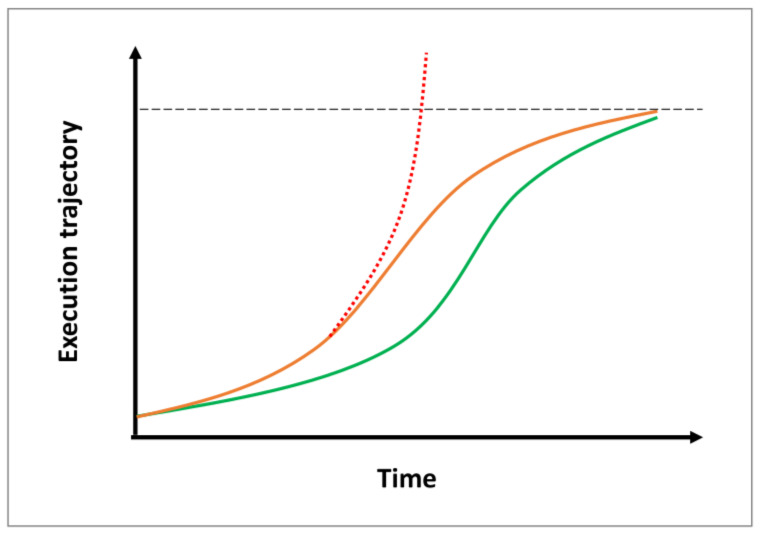
Proposed model of vimentin as a context-dependent regulator that accelerates biological processes during progression while decelerating them during resolution. The temporal trajectory of a representative biological process, e.g., wound healing, in the presence (orange) and absence (green lines) of vimentin. The level of process completion (horizontal dashed line), and the accelerated process that persists beyond completion under pathological conditions (dotted red line), are shown, such as wound over-repair leading to, e.g., fibrosis.

We propose a conceptual model (Figure 2) in which vimentin enables biological systems to optimize their responses to adversity by dynamically amplifying counteracting feedback mechanisms yet also raise the potential for maladaptive overactivation when regulatory constraints fail.

#### 3.4.1. In Physiology

Vimentin functions as a facilitator, that amplifies stimulatory signals to accelerate the progression by providing positive feedback after a system’s initial response to insult, and amplifies subsequently activated inhibitory signals, through negative feedback, to ensure a controlled resolution. Vimentin can thereby narrow the window of vulnerability to ensures robust threat-neutralization and a timely, smooth return to homeostasis (Figure 2).

#### 3.4.2. In Pathology

When an insult persists, such as refractory infection in sepsis or chronic tissue injury in fibrosis, the underlying signals remain active. In its role as a faithful gain-of-function amplifier, vimentin relentlessly intensifies stimulatory responses to neutralize the threat, and fails to switch off. When this amplification surpasses the compensatory capacity of inhibitory mechanisms, the system overshoots its regulatory threshold, driving further dysregulation (Figure 2).

## 4. The Tipping Point: From Physiological Adaptation to Pathological Collapse

### 4.1. The Cost of Amplification: Disequilibrium Because of Defense

As a signal amplifier, vimentin strengthens system resilience against external insults. By enhancing signal gain, it permits the necessary excursion from homeostasis required to mount a robust defense. This explains why vimentin-deficient animals survive in protected environments but fail when challenged [15,35].

However, this amplification generates instability. Because it operates by pushing the system away from equilibrium, intense or persistent insults can cause vimentin-amplified signaling to surpass manageable limits. What begins as a functional adaptive response marked by controlled instability devolves into a pathological reaction, causing disruptive disequilibrium and unsustainable fragility. Here, vimentin paradoxically transforms from a guardian of resilience into the primary driver of systemic failure.

### 4.2. A Conceptual Model of Systemic Failure

**Figure 3:**
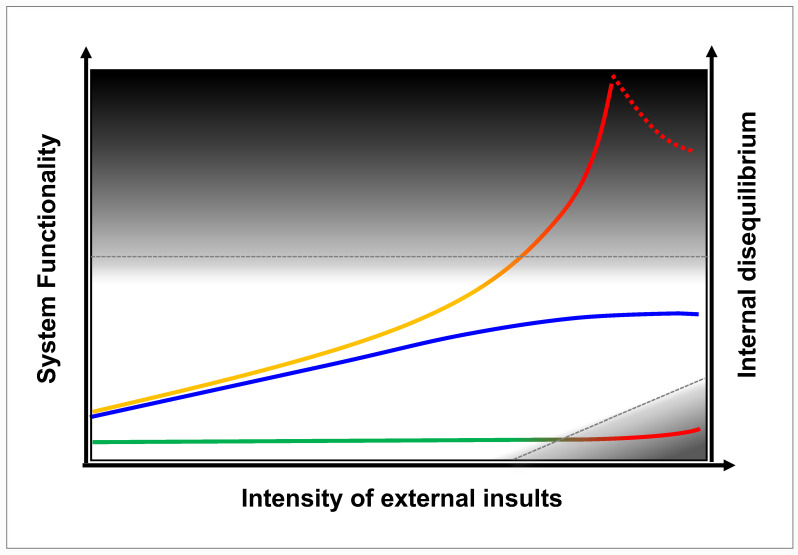
Conceptual schematic of vimentin as a system fate determinant in response to external insult: amplifying functional responses to provide resilience and exacerbating internal disequilibrium to precipitate pathological collapse. States of a system: health (white) or disease (dark zone); the roles of vimentin: absent (lower green/red line), modulated (middle blue line), and uncontrolled (upper orange/red line); system collapse: dying (dotted segment of upper line); red segments of lines: maladaptive functionality and disruptive disequilibrium (upper line), or insufficient functionality (lower line); transitions between health and disease: threshold (dashed gray lines).

We propose a unified conceptual model (adapted from [72]) that maps the evolving trajectory of health states in a complex biological system (Figure 3).

#### 4.2.1. System States Under External Perturbations

Health (White Zone): Represents homeostasis or effective adaptation. This includes systems with basal function and minimal disequilibrium under mild stress (Figure 3 lower trajectory, green segment), as well as systems mounting an enhanced, adaptive response (medium levels of functionality and internal disequilibrium) under moderate-to-intense stress (middle and upper trajectories, left portion). Notably, modulated vimentin activity (middle blue trajectory) preserves this healthy state even under intense external insults.

Disease (Dark Zone): Represents systems failure via two distinct pathways: (1) Insufficiency, where a system with basal function in homeostatic state is overwhelmed by severe external insults (lower trajectory, red segment in dark zone); (2) Maladaptation, where uncontrolled vimentin activity drives excessively high functionality and disruptively high internal disequilibrium (upper trajectory, solid red segment in dark zone), eventually leading to systems collapse (upper trajectory, dotted red segment in dark zone).

#### 4.2.2. Vimentin as a Determinant of Systems-Level Outcomes

The Basal State: Without vimentin, the system maintains homeostasis and basal functions adequately under mild stress but collapses under severe insults due to an inability to mount a sufficient response. This represents a state of fragility (**Rigid Failure**).

The Uncontrolled State: With unchecked vimentin as an amplifier, the host system mounts a massive response. While this amplified response provides high functional capacity, it generates excessive internal disequilibrium. The functionality of a system and its internal disequilibrium are interconnected and interdependent. Under severe and persistent stress, this vimentin-dependent amplification becomes self-reinforcing, propelling the system into a maladaptively high functionality and a destabilizing high disequilibrium that, once pushed beyond its critical stability threshold, leads to systemic breakdown (**Chaotic Collapse**).

The Modulated State: A therapeutic goal is to obtain an optimal trajectory, in which vimentin can be modulated to provide sufficient amplification for threat neutralization while maintaining disequilibrium within a manageable, resilient range (**Adaptive Resilience**, middle trajectory across the entire white zone).

This model provides a unified framework to reconcile conflicting observations that vimentin may be beneficial, harmful, or dispensable, reflecting the system’s dynamic requirements under external perturbations.

#### 4.2.3. Sepsis as a Crisis of Internal Regulation

Crucially, this model reframes the etiology of sepsis. The disease arises not merely from the external pathogen load, but from the host’s inability to manage the internal disequilibrium generated by its own amplified defense systems. As applied to sepsis, this framework elucidates the “uncoupling” phenomenon: when vimentin excessively amplifies antagonistic pathways, simultaneously driving hyperinflammation and immunosuppression, the tension between these opposing forces eventually exceeds what the system’s structural integrity can tolerate. The result is a catastrophic divergence, where the patient suffers from concurrent hyperinflammation and immune paralysis. Thus, the coexistence of these opposing pathologies is not a paradox, but the inevitable consequence of an over-amplified system in collapse.

## 5. Vimentin as the Architect of Sepsis Dysregulation

As described above, sepsis is characterized by the simultaneous collapse of diverse biological systems, encompassing immunological, vascular, coagulative and metabolic functions. Vimentin’s strategic localization at the nexus of these networks positions it as a unique systems-level target. The evidence below suggests that vimentin does not merely participate in these cascading failures; rather, it actively orchestrates them.

### 5.1. Innate Immunity: Driving the Cycle of Hyperinflammation and Immune Paralysis

Innate immune cells are the primary drivers of sepsis pathology. Vimentin acts as a “double agent” within cells and systemically through its extracellular release, amplifying both the initial cytokine storm and the subsequent immune paralysis.

#### 5.1.1. Neutrophils

In early sepsis, vimentin is critical for pathogen detection and clearance. It acts as an endogenous ligand for Pattern Recognition Receptors (PRRs), including toll-like receptors (TLRs), nucleotide-binding oligomerisation domain-containing protein 2 (NOD2) and Dectin-1, facilitating rapid threat detection [73,74,75]. Additionally, vimentin physically orchestrates nuclear segmentation and cytoskeletal remodeling to enable neutrophil swarming and phagocytosis [76,77]. However, this amplification becomes increasingly more maladaptive as sepsis progresses. Vimentin sustains the release of lytic factors such as reactive oxygen species (ROS) and neutrophil extracellular traps (NETs), which damage host tissue while simultaneously restraining mitochondrial function or ROS production needed for microbial killing [73,77]. This creates a paradox where neutrophils are hyper-destructive to the host yet hypo-effective against the pathogen.

#### 5.1.2. Macrophages

Macrophage exhibits a spectrum of activation states. Its plasticity, particularly the ability to transition between pro-inflammatory M1 and pro-healing M2 phenotypes, is essential for effective host defense, enabling pathogen clearance followed by tissue repair. The M1 and M2 phenotypes exhibit distinct morphologies: M1 macrophages are typically round or flattened, whereas M2 macrophages adopt an elongated shape. Vimentin provides the structural machinery required for this elongation [16]. Remarkably, cell geometry alone is sufficient to bias macrophage fate, with elongation promoting M2 marker expression while suppressing inflammatory M1 cytokine secretion [78].

Beyond morphology, vimentin stabilizes CD11b at the surface of M2 macrophages, enabling podosome formation and extracellular matrix degradation required for tissue remodeling [79]. Conversely, vimentin is structurally and functionally required for nitric oxide (NO) production [80], a defining feature of activated M1 macrophages. Vimentin serves as a scaffold for NLRP3 inflammasome assembly, driving IL-1β release and acute lung injury [19]. While amplifying inflammatory signaling to support pathogen clearance, vimentin simultaneously compromises antimicrobial defense. Vimentin sequesters the p47Phox subunit of NADPH oxidase, impairing the oxidative burst required for effective bacterial killing [44]. In parallel, vimentin physically interacts with the kinases TBK1 and IKKε, preventing their association with interferon regulatory factor 3 (IRF3), thereby inhibiting IRF3 phosphorylation and nuclear translocation and consequently, suppressing type I interferon production, a critical antiviral defense mechanism [81].

Together, these findings establish vimentin as a bidirectional regulator of macrophage polarization and effector function. However, in pathological contexts such as sepsis, this regulatory capacity becomes maladaptive. As a result, vimentin can drive macrophages into a dysfunctional hybrid state, simultaneously hyperinflammatory and immunologically ineffective, thereby amplifying tissue damage while compromising pathogen clearance.

### 5.2. Adaptive Immunity: Fueling Inflammation and Promoting Immunosuppression

The transition from early hyperinflammation to subsequent immunosuppression is also contributed by an imbalance between pro-inflammatory Th17 cells and immunosuppressive regulatory T cells [82,83].

#### 5.2.1. Th17/Treg Imbalance

Th17 and Treg cells function as a reciprocal counterbalancing pair; their dynamic shift in dominance dictates the ‘bloom and wane’ of the host inflammatory response throughout sepsis [83]. Vimentin acts as a pivotal regulator of this axis. It is a potent driver of type 17 immunity: exposure to vimentin significantly expands the Th17 population and amplifies IL-17 production [84,85]. Conversely, vimentin modulates Treg function through dual mechanisms: intracellular vimentin restrains Treg suppressive activity via the distal pole complex [18,86], while extracellular vimentin acts as a signal that triggers an immunosuppressive cascade, creating a tolerogenic environment where Tregs can thrive or exert their suppressive function [87]. In early sepsis, Th17 dominance drives hyperinflammation and tissue damage, whereas in the late phase, the exhaustion of Th17 and the expansion of highly active Tregs swings the system toward immune paralysis, a state strongly associated with lethal secondary infections [88,89,90]. Crucially, this biphasic pattern can uncouple in severe sepsis. In these patients, Th17 hyperactivity can occur concurrently with Treg expansion [91], resulting in the paradoxical and lethal coexistence of hyperinflammation and immunosuppression.

#### 5.2.2. Unique Therapeutic Opportunity

Modulating vimentin presents a rare systems-level intervention capable of acting across phases of sepsis pathobiology, mitigating the acute Th17-meadiated hyper-inflammatory response while preventing the progressive, Treg-mediated immunosuppression that characterizes late-stage immune dysfunction. This dual capacity makes vimentin modulation a promising strategy to treat the complex clinical phenotype of concurrent hyperinflammation and immunosuppression.

### 5.3. Systemic Collapse: Vascular, Coagulative, and Metabolic Failure

Beyond immunity, vimentin creates a “perfect storm” of organ dysfunction by dysregulating multiple other critical systems.

#### 5.3.1. Vascular Leak and Shock

Septic shock emerges when vascular leakage converges with uncontrolled vasodilation and impaired vascular tone, resulting in refractory hypotension that persists despite fluid resuscitation [92,93]. In endothelial cells, extracellular vimentin engages VEGFR2, activating signaling cascades that dismantle endothelial barrier integrity and drive profound plasma extravasation [94,95]. Concurrently, cytoplasmic vimentin is required for stress-induced nitric oxide (NO) production and vascular smooth muscle relaxation [80]. Excessive NO generation promotes unrestrained vasodilation and further disrupts endothelial barriers, together producing the severe, unresponsive hypotension that defines septic shock [96,97].

#### 5.3.2. Coagulation and Thrombosis

Sepsis is plagued by Disseminated Intravascular Coagulation (DIC), a life-threatening complication characterized by systemic, dysregulated activation of the coagulation cascade, leading to both thrombosis and bleeding [8]. Platelets, von Willebrand Factor (VWF), fibrinogen, and Tissue Factor (TF) orchestrate the transition from a primary platelet aggregates to a mature fibrin-rich thrombus. Vimentin bridges platelets to VWF and interacts with fibrinogen and TF, fueling microvascular thrombosis that starves organs of oxygen [22,23,98,99,100].

#### 5.3.3. Metabolic and Autophagic Stress

Sepsis induces profound metabolic reprogramming, disrupting glucose, lipid, and protein homeostasis across tissues and disease phases. While systemic catabolism dominates, driving global breakdown of energy stores, certain tissues paradoxically engage in localized anabolism to amplify immune and repair functions [9,10]. Vimentin emerges as a critical node in the linking of oxidative stress to metabolic regulation. Notably, vimentin depletion mitigates hyperglycemia and insulin resistance [101], which are hallmarks of septic metabolism that persist despite nutrient deficiency due to cytokine-driven disruption of insulin signaling [102]. At the molecular level, vimentin promotes hyperglycemia by sequestering the Beclin-1/14-3-3 complex, thereby inhibiting autophagy [54,101,103]. Consequently, the depletion or degradation of vimentin releases this brake, enhancing autophagic flux and restoring metabolic fitness. While dysregulated autophagy can spiral into uncontrolled cell death [104], modulating this vimentin-dependent checkpoint offers a promising strategy to resolve sepsis-induced metabolic failure.

### 5.4. The Systems-Level Solution: Reconciling the Irreconcilable

**Figure 4:**
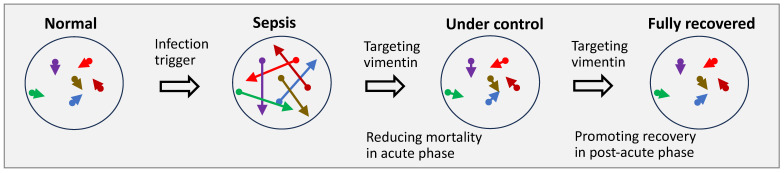
Conceptual schematic of vimentin modulation as a systems-level strategy to counter sepsis: restoring network logic to restrain multiple pathological processes, prevent systemic collapse, and promote recovery. Arrows with different colors represent diverse vimentin-linked processes on cellular, tissue, organ, system or organism-levels that are dysregulated in sepsis, such as inflammation, immunosuppression, coagulation, hemorrhage, anabolism, and catabolism. The arrow lengths indicate magnitudes of activities; and the divergent arrow directions, interrelationships and inter-dependency of these biological processes.

The historical failure of reductionist therapies lies in their inability to simultaneously address the opposing processes within sepsis. A drug that blocks inflammation risks exacerbating immune paralysis, while one that boosts immunity may worsen septic shock. Vimentin modulation offers a fundamentally different paradigm. Because vimentin functions as a context-dependent amplifier rather than a binary switch, targeting it does not simply “block” a pathway. Instead, it modulates the biochemical and biophysical gain of the network, which is formed by vimentin and its interactome where vimentin provides an on-demand biophysical platform that enhances the biochemical performance of its binding partners. This unique mechanism allows for the simultaneous attenuation of hyperinflammation and the re-engagement of pathogen clearance. Consequently, vimentin-targeted therapy acts as a system-wide stabilizer, capable of harmonizing the discordant signals across infection, immune dysregulation, endothelial dysfunction, coagulopathy, and metabolic disruption that collectively drive sepsis mortality (Figure 4).

## 6. From Theory to Therapy: Evidence for Vimentin Modulation in Sepsis

### 6.1. Clinical Validation: Vimentin Is Beyond a Prognostic Signal

Vimentin has emerged as a critical biomarker of sepsis severity. Circulating levels of extracellular vimentin are markedly elevated in septic patients, correlating strongly with poor prognosis and increased mortality [105,106]. This elevation in extracellular vimentin may not merely be a bystander effect just for serving as a prognostic marker; rather, it contributes to immune dysregulation. Extracellular vimentin functions as a DAMP that amplifies innate inflammation (e.g., via TLR4 on neutrophils) while also modulating antigen-presenting cells and vascular/stromal niches in ways that can favor immune suppression [73,87,107]. Mechanistically, inflammatory stress triggers the secretion of vimentin via exosomes [108]. Since modulating intracellular vimentin has been shown to attenuate this exosomal release *in vivo* in mice [109], therapeutic targeting of vimentin may offer a strategy to sever this self-perpetuating cycle of systemic dysfunction.

### 6.2. The Statin Paradox: A Retrospective Clue

The failure of drug repurposing strategies in sepsis offers compelling, albeit indirect, support for vimentin as a target. Statins (HMG-CoA reductase inhibitors) were extensively evaluated for their anti-inflammatory potential but failed to reduce mortality of sepsis patients in broad meta-analyses [110,111]. However, a granular analysis uncovers a striking divergence: while Rosuvastatin was associated with worse outcomes, likely due to its detrimental cholesterol lowering in sepsis, Simvastatin exhibited a trend toward improved survival [112]. The explanation likely lies in off-target pharmacology: unlike other statins, Simvastatin uniquely binds and modulates vimentin [113]. This suggests that the failure of Simvastatin in clinical trials for sepsis may not necessarily reflect a failure of the underlying mechanism but a limitation of specificity; its survival benefit via vimentin modulation was likely negated by the deleterious effects of lipid suppression. Taken together, this implies that a selective vimentin modulator, uncoupled from cholesterol metabolism, could unmask this latent therapeutic efficacy.

### 6.3. ALD-R491: Validating the Systems-Level Approach

While tool compounds like Withaferin A have validated vimentin’s therapeutic potential, their lack of specificity and suboptimal bioavailability have hindered clinical translation [114,115]. To address these barriers, a specific vimentin modulator with desired druggable profiles is required. The small molecule compound ALD-R491 is a highly specific vimentin modulator discovered through phenotypic screening and validated by affinity-based proteomics, mutagenesis studies, and off-target profiling [72,116,117]. Subsequent preclinical studies with ALD-R491 suggest that the specific vimentin modulation could confer adaptive resilience across cellular, tissues, organs, and the whole organism levels, without detectable toxicity.

#### 6.3.1. Restoring Immune Balance

ALD-R491 was shown to enhance macrophage pathogen clearance by releasing vimentin-sequestered p47Phox, thereby boosting bacterial killing, while simultaneously to activate Tregs by dismantling the vimentin dependent Distal Pole Complex (DPC) [118], a mechanism that may help mitigate hyperinflammation in the early stages of sepsis. Interestingly, ALD R491 was shown to completely block IL 2 and TGF-β-induced Treg differentiation (Aluda, unpublished data), suggesting that this compound might prevent the expansion of highly activated Tregs in late-stage sepsis [91,119] and thereby avert immune paralysis.

#### 6.3.2. Resolving Tissue Damage and Organ Failure

In CLP-induced sepsis models, ALD-R491 significantly reduced systemic inflammation, preserved lung, liver, and kidney function, and improved survival, both as a monotherapy and in combination with antibiotics [120]. Its tissue protective effects have also been demonstrated *in vivo* across multiple disease models induced by chemical or biological insults [72,118].

#### 6.3.3. Broad Efficacy

Beyond sepsis, the compound was shown to have *in vivo* efficacy in animal models of fibrosis, bacterial and viral infection (SARS-CoV-2), and auto-inflammatory disease [72,118,120], suggesting that targeting vimentin could address the shared logic of complex host response diseases.

#### 6.3.4. A New Path Forward

The consistent efficacy of vimentin modulation across diverse models of injury and infection suggests that the apparent chaos of sepsis reflects not insurmountable randomness, but a systems-level dysregulation amenable to targeted, network-based intervention. By shifting focus from pathogen elimination to host response realignment, vimentin-targeting strategies offer the first viable path to treating the “intractable” complexity of sepsis.

## 7. Sepsis’s Inherent Complexity Demands a Paradigm Shift to Systems-Level Drug Development

### 7.1. The Reductionist Fallacy: Why Current Paradigms Fail

Sepsis represents a catastrophic destabilization of complex biological systems, characterized by nonlinear dynamics, decentralized control, and the uncoupling of antagonistic pathways. This fundamental biology distinguishes sepsis from many other diseases and demands a departure from prevailing therapeutic dogmas. Despite decades of investment, sepsis drug development remains confined to reductionist paradigms, constrained by conceptual limitations as much as by technological ones, a reality increasingly acknowledged in the literature [6,10,121,122,123].

#### 7.1.1. The Antibiotic Ceiling

Sepsis is frequently misconstrued as a purely pathogen-driven condition, fostering an overreliance on broad-spectrum antibiotics. Although indispensable for pathogen clearance, antibiotics offer no coverage against many causative microorganisms, including viruses, fungi, and parasites, and their effectiveness is further undermined by antimicrobial resistance and unidentified pathogens. More fundamentally, once the inflammatory cascade is initiated, pathogen-directed therapies cannot halt the self-perpetuating dysregulation of the host response [124,125]. As sepsis progresses from infection toward systemic collapse, the therapeutic leverage of pathogen control rapidly diminishes, leaving patients vulnerable to injury driven primarily by their own dysregulated host response.

#### 7.1.2. The Nonlinear Trap

Early detection-intervention frameworks, including the Surviving Sepsis Campaign, are largely built on linear progression models that fail to capture the nonlinear, chaotic dynamics of sepsis. Because the syndrome is driven by nonlinear amplification, patients can transition from apparent stability to rapid collapse within minutes [126]. This intrinsic unpredictability renders “early” intervention windows inherently unstable and difficult to define. Moreover, most sepsis-related deaths occur in patients with substantial comorbidities, and only 12% are considered preventable even under optimal care [127]. Although hospital process improvements, such as earlier antibiotic administration and standardized sepsis bundles, have improved outcomes, further refinement now yields diminishing returns, indicating that hospital-based interventions alone are approaching their ceiling of effectiveness.

#### 7.1.3. The Snapshot Fallacy

Precision medicine presupposes stable molecular drivers; sepsis has none. Its pathogenesis is decentralized, nonlinear, and continuously reconfigured over time. Molecular endotypes therefore capture only transient snapshots of a storm in motion [128]. Therapeutics built around fixed biomarkers are fundamentally mismatched to the unpredictable dynamics of disease progression because targets shift before interventions can take effect, and no single marker exercises dominant regulatory control. Nevertheless, most contemporary sepsis drug-development efforts continue to pursue this paradigm [129,130], despite repeated clinical trial failures and, in some cases, demonstrable harm, across biomarker-driven strategies [131,132,133,134,135,136,137,138]. Ignoring the nonlinear, time-dependent dynamics of sepsis and relying on single-time-point biomarker stratification is therefore unlikely to yield effective therapies.

#### 7.1.4. The Uncoupling Problem

The alternating or simultaneous manifestation of opposing pathological states in sepsis, such as hyperinflammation and immunosuppression, microvascular coagulation and hemorrhage [6,7,8,13], reflect a system that has lost its capacity to regulate bidirectional processes. Therapeutic attempts to suppress one arm of these polarized states invariably exacerbate the other, highlighting a fundamental mismatch: linear, unidirectional interventions cannot resolve a nonlinear, multidimensional systems failure.

### 7.2. The Systems Strategy: Vimentin as a Determinant of System Fate

Resolving sepsis requires shifting from the static question of *what* is elevated to the dynamic inquiry of *how* the system is regulated. This reframing redirects therapeutic focus away from managing downstream manifestations and toward correcting the core mechanistic logic underlying system collapse. Within this framework, vimentin’s function as a regulatory network hub is uniquely compelling: rather than regulating one or a few isolated pathways, targeting vimentin offers the potential to re-establish coordinated, bidirectional control across the interdependent systems that fail in sepsis.

#### 7.2.1. Vimentin as the Architecture of Instability

From a systems-biology perspective, sepsis emerges when the ‘gain’ of host-defense mechanisms is driven beyond physiological limits, leading to fracture of regulatory feedback loops. Vimentin functions as a central gain controller (or amplifier) within this architecture. Although classically viewed as a structural protein, vimentin serves as a network-hub position that integrates mechanical cues with biochemical signaling to coordinate stress responses. In health, vimentin generates a necessary tension, amplifying defense programs just enough to neutralize threats while preserving the system integrity. In sepsis, however, this amplification becomes persistent and unrestrained. Vimentin drives the system into an unsustainable state of high-tension disequilibrium or instability, precipitating catastrophic uncoupling of protective and regulatory processes, and consequently, systemic collapse. Therapeutically modulating vimentin therefore offers a rare systems-level intervention: by attenuating excessive signal amplification across the network, it permits endogenous counter-regulatory mechanisms to re-engage and restore stability.

#### 7.2.2. Pan-Endotype Efficacy

Because vimentin modulation targets the shared machinery of the host response rather than a specific trigger, it obviates the need for precise molecular stratification. In sepsis, a syndrome where heterogeneity arises from dynamic progression rather than static molecular aberrations, vimentin modulation offers the potential for pan-endotype efficacy. By acting as a systems-level regulator, vimentin modulation can function as a universal stabilizer, applicable across the chaotic spectrum of sepsis phenotypes.

#### 7.2.3. Inherent Safety

Unlike enzyme-targeting therapeutics (e.g., kinase inhibitors), vimentin-modulating agents do not suppress essential biochemical activities. Instead, they temper the dynamics of the filamentous scaffold that amplifies signaling across the network. Whereas deep inhibition of critical enzymes predictably produces toxicity, reducing vimentin’s filament dynamics constrains pathological signal amplification without affecting the basal functions of its interacting partners. Importantly, vimentin modulators neither degrade the protein nor directly reduce its abundance; even complete genetic ablation of vimentin yields organisms that remain viable, albeit less optimally adapted. Consequently, modulating vimentin to prevent the system from tipping into **Chaotic Collapse** is unlikely to induce **Rigid Failure**. Rather, it preserves the system within an optimal regime of flexible stability, an adaptive equilibrium often termed flexstability or **Adaptive Resilience** (Figure 3). Both mechanistic rationale and available preclinical and clinical data support the favorable safety profile of vimentin modulation.

#### 7.2.4. A Universal Principle for Complex Disease

Sepsis represents the most extreme manifestation of a general biological failure: the transition of adaptive responses into maladaptive pathology under conditions of persistent or unresolved insult. The same vimentin-driven amplification logic underlies other complex diseases, including autoimmune disorders (unchecked immune activation) and fibrosis (unchecked tissue repair). Thus, successful therapeutic modulation of vimentin in sepsis would not only address a long-standing clinical crisis but also validate a broader paradigm for treating complex diseases, i.e., targeting the logic of system dysregulation rather than its downstream symptoms.

### 7.3. Breaking the Impasse in Sepsis: From Reductionism to Systems-Oriented Innovation

True innovation in drug development is frequently constrained by systemic barriers, including institutional inertia, conservative investment strategies, and rigid regulatory frameworks. Nowhere is this more apparent than in sepsis. Despite profound unmet need, therapeutic development in this field has suffered extensive divestment by major pharmaceutical companies and institutional investors. A history of unsuccessful trials has rendered transformative breakthroughs seemingly implausible, driving a retreat toward lower risk, incremental approaches. As a result, the therapeutic pipeline has effectively stagnated, with efforts shifting to drug repurposing, which prioritizes safety over efficacy, or toward refinement of diagnostics rather than mechanistic innovation.

Although small biotechnology companies retain the agility to pursue disruptive ideas, their capacity is severely constrained by the scarcity of capital in such a high-risk landscape. Overcoming this stagnation requires a fundamental conceptual pivot: abandoning reductionist assumptions and recognizing sepsis as a nonlinear, systems-level collapse. The apparent chaos of sepsis reflects not insurmountable randomness, but a systems-level dysregulation amenable to targeted, network-based intervention. While the concept remains to be clinically proven, by shifting focus from pathogen elimination to host response realignment, vimentin-targeting strategies offers the most plausible route to reinvigorating the pipeline and delivering truly transformative care for sepsis.

## 8. Conclusions

Sepsis reflects the **Chaotic Collapse** of coordinated biological systems rather than dysfunction within any single signaling pathway. Its inherent complexity therefore necessitates an integrative, systems-oriented therapeutic framework, moving beyond reductionist approaches that target isolated pathogenic components. Vimentin functions as a central signaling hub, integrating and propagating host responses to external insults across interconnected cellular networks. Under sustained or excessive stress, this adaptive role could shift toward maladaptive dysregulation, uncoupling normally synchronized cellular programs, destabilizing network architecture, and precipitating systems failure. Modulating vimentin thus could enable a systems-level intervention, simultaneously realigning counteracting biological processes to preserve system integrity and restore homeostasis. Supported by a strong mechanistic rationale, robust preclinical efficacy, and emerging clinical signals, vimentin modulation represents a promising network-realigning therapeutic paradigm with the potential to overcome the translational limitations that have long constrained sepsis drug development.

## Data Availability

No new data were created or analyzed in this study. Data sharing is not applicable to this article.
